# The pain-comorbidity links: a cross-sectional analysis of musculoskeletal burden in Saudi older adults

**DOI:** 10.3389/fpubh.2026.1727241

**Published:** 2026-01-21

**Authors:** Hammad S. Alhasan, Mansour Abdullah Alshehri, Aqeel M. Alenazi

**Affiliations:** 1 Department of Medical Rehabilitation Sciences, Faculty of Applied Medical Sciences, Umm Al-Qura University, Makkah, Saudi Arabia; 2 Department of Health and Rehabilitation Sciences, College of Applied Medical Sciences, Prince Sattam Bin Abdulaziz University, Al-Kharj, Saudi Arabia

**Keywords:** diabetes, hypertension, musculoskeletal pain, obesity, older adults, Saudi Arabia

## Abstract

**Background:**

Musculoskeletal pain and noncommunicable diseases are major contributors to disability among adults aged ≥50 years, yet their coexistence and modifying factors are not well characterized in Saudi Arabia. This study aimed to estimate age-group–specific prevalence of musculoskeletal pain, identify independent predictors of site-specific and multisite pain and of major morbidities, and evaluate effect modification among adults ≥50 years.

**Methods:**

A community-based cross-sectional survey was administered to participants aged ≥50 years. Data on sociodemographic characteristics, smoking status, body mass index (BMI), morbidities, and musculoskeletal pain sites were collected.

**Results:**

Among 298 participants (mean age 58.2 ± 6.3 years; 47.0% overweight; 32.9% obese), musculoskeletal pain was reported by 73.2%, most commonly at the lower back (30.9%), knee (26.2%), neck (23.8%), and shoulder (21.8%). Hypertension (29.5%) and diabetes (28.2%) were the most prevalent morbidities; multimorbidity was present in 24.5% of participants. Female sex was the most consistent predictor across pain sites, with higher odds for low back pain (OR 2.83, 95% CI 1.60–5.00; *p* < 0.001), shoulder pain (OR 2.99, 95% CI 1.64–5.44; *p* < 0.001), and neck pain (OR 2.58, 95% CI 1.44–4.63; *p* = 0.002). A significant smoking × BMI interaction was observed for hypertension (OR 0.22, 95% CI 0.06–0.86; *p* = 0.029), indicating that the positive association of smoking with hypertension was strongest at normal BMI and attenuated at higher BMI. For diabetes, the age-related increase in risk was greater among participants with hypertension (OR 1.02 per year, 95% CI 1.01–1.03; *p* < 0.001) and was further increased at higher BMI (OR 1.59, 95% CI 1.24–2.05; *p* < 0.001).

**Conclusion:**

Musculoskeletal pain co-occurring with cardiometabolic conditions was common among older adults in Saudi Arabia. Female sex consistently predicted pain, and obesity was more strongly associated with hypertension. Effect-modification patterns identify high-risk strata; therefore, routine cardiometabolic screening should be incorporated into musculoskeletal management, with first-line non-pharmacologic care and targeted counselling for obese women and smokers.

## 1 Introduction

Musculoskeletal pain and common noncommunicable diseases (e.g., hypertension, diabetes, and dyslipidaemia) are major determinants of disability and healthcare utilization in older adults (1, 2). Global Burden of Disease (GBD) analyses consistently rank musculoskeletal conditions among the leading causes of disability worldwide, with low back pain being the leading contributor to nonfatal disability. Noncommunicable diseases likewise account for the largest share of disability burden globally (1). At the same time, changes in lifestyle behaviours are modifying risk patterns in older adults. International surveillance reports indicate persistently high levels of physical inactivity, a behaviour strongly associated with cardiometabolic risk, functional decline, and musculoskeletal pain (3, 4). The GBD framework highlights a growing population-level impact of metabolic risks, particularly high body mass index (BMI) and elevated fasting plasma glucose, underscoring the importance of prevention strategies that address clusters of interrelated risk factors rather than single conditions in isolation (5, 6). These overlapping risk patterns contribute to increased healthcare demand and reduced quality of life among older adults.

In Saudi Arabia, rapid population aging, high obesity prevalence, and altered health behaviours highlight the need for epidemiological evidence mapping site-specific and widespread musculoskeletal pain in conjunction with major morbidities, while examining whether sociodemographic factors modify these associations (7–9). Recent national reports show ongoing development toward the life-expectancy target (increasing from 74 years to 78.8 years in 2024), highlighting the importance of preventing and managing chronic conditions that limit healthy lifespan, including musculoskeletal pain and noncommunicable diseases. In parallel, national surveillance demonstrates persistent lifestyle-related risks, particularly unhealthy dietary patterns and physical inactivity, reinforcing the need to characterize how lifestyle factors interact with musculoskeletal pain and multimorbidity in older adults (10). Recent syntheses confirm that musculoskeletal pain, especially low back pain, dominates nonfatal disability outcomes, whereas emerging studies indicate that multisite pain, rather than single-site pain, is common and clinically significant (11, 12). Clinically, multisite pain is associated with greater disability, increased risk of fall, poorer quality of life, and increased healthcare use compared with localized pain (13–15). Evidence from a large cohort study demonstrates a consistent pattern of co-occurring pain, and epidemiological evidence links multisite pain with outcomes beyond pain symptoms, including cardiometabolic events and cognitive decline (16). Epidemiological data indicate that chronic pain is prevalent in adults in Saudi Arabia, with a national survey estimating that chronic pain affects 46%, with low back pain being the most prevalent site and higher prevalence observed among female participants and older adults (17). Regional burden data from the Gulf Cooperation Council further demonstrate a high prevalence of musculoskeletal disorders in Saudi Arabia (19% in 2019), highlighting musculoskeletal conditions as a long-term public-health concern (18).

Community- and setting-specific studies similarly report multiple affected pain regions, with the low back being most frequent, and worse outcomes in females and older adults (19, 20). However, population-(17) based data focusing on older adults (≥50 years) remain scarce in Saudi Arabia, limiting age-relevant risk stratification. Epidemiological studies in Saudi Arabia align with international findings showing the interplay between metabolic conditions and musculoskeletal outcomes. For instance, in adults with knee osteoarthritis, higher BMI has been associated with worse physical function, reflecting a metabolic-mechanical pathway (21). Furthermore, among older adults, BMI patterns have been linked to neurological and musculoskeletal disorders, reinforcing obesity-related risks in late life (22).

Conceptual frameworks emphasize mechanical loading, metabolic-inflammatory processes, and central sensitization mechanisms (23, 24), suggesting multiple prevention targets and potentially varied risk patterns across sex and BMI categories. Evidence that multisite pain is common and linked to worse outcomes than localized pain, combined with the observation that metabolic risks accumulate with age, highlights the need for interaction-focused epidemiological analyses in older adults. However, the majority of existing datasets concentrate on single pain sites, are restricted to clinical populations, or do not evaluate whether associations differ across key subgroups (25). Because musculoskeletal pain and cardiometabolic conditions likely interact through shared mechanical, inflammatory, vascular, and behavioural mechanisms, an interaction-cantered approach may reveal hidden heterogeneity and identify subgroups at heightened risk.

In spite of the growing body of work, three important gaps remain. First, most studies in Saudi Arabia rely on setting-specific or convenience samples, limiting generalizability to older adults. Second, while multisite pain is often reported, it is rarely included in predictive models, leaving its prognostic value insufficiently understood. Third, few studies examine effect modification (e.g., sex × BMI) or apply interaction models capable of identifying high-risk subgroups for targeted interventions. Therefore, the objective of the current study was to generate evidence that can guide policy and clinical practice in alignment with Saudi Arabia’s Vision 2030, which targets an increase in healthy life expectancy. This study examines: (i) prevalence and age-group differences (50–60 vs. ≥61); (ii) independent predictors of musculoskeletal pain sites and multisite burden; (iii) independent predictors of major morbidities; and (iv) potential effect modification by key variables.

## 2 Materials and methods

### 2.1 Study design

A community-based, cross-sectional study was conducted among older adults residing in Saudi Arabia. Data were collected via an online survey distributed during the study period using convenience sampling. Ethical approval was obtained from the Biomedical Ethics Committee of Umm Al-Qura University, Saudi Arabia (Approval no.: HAPO-02-K-012-2024-12-2352). The study adhered to the ethical principles of the Declaration of Helsinki and was reported in accordance with the Strengthening the Reporting of Observational Studies in Epidemiology (STROBE) guidelines.

### 2.2 Participants

Participants were adults aged ≥50 years residing in Saudi Arabia. Eligibility criteria required that participants be able to read and respond to the online survey autonomously or with minimal assistance. Participants younger than 50 years were excluded. Informed consent was obtained electronically before participation. Participation was completely voluntary, data protection and participant anonymity were ensured throughout data collection and analysis.

### 2.3 Eligibility criteria

Participants were eligible if they were aged ≥50 years and residing in Saudi Arabia; had no history of major injuries or surgeries. Participants not meeting these criteria were excluded from the study.

### 2.4 Data collection

Data were collected via an online survey hosted on SurveyMonkey.^1^ The survey link was shared via social media platforms and community channels, and it was available for a five-month period from December 2024 to April 2025. A helpline (phone, WhatsApp, or email) was available for technical support, and offered the option of phone-assisted survey completion using a standardized script if needed. Electronic informed consent was obtained after participants reviewed the initial survey page, which outlined the study’s objectives, procedures, confidentiality safeguards, and participants’ right to withdraw. All responses were stored securely in a password-protected database accessible only to the research team.

### 2.5 Survey

The survey was designed to minimize response bias by presenting items in a standardized sequence and covered three main domains:

1. Sociodemographic variables

This part of the survey focused on collecting demographic and eligibility characteristics to facilitate interpretation of the findings and control for confounding factors. Data were collected on age in years, sex, nationality, region of residence, marital status, living arrangements, educational level, employment status, work environment, and average daily work/study hours. Self-reported height and weight were obtained to calculate BMI. Smoking status was also recorded. Participants were asked about musculoskeletal pain during the past week, prior major injuries or surgeries, and the presence of chronic or serious medical conditions.

2. Musculoskeletal pain

Participants were asked whether they had experienced musculoskeletal pain. If pain was reported, they were asked to specify the anatomical regions affected using a list that included head, neck, thoracic spine, lower back, sacroiliac region, pelvis, abdomen, shoulder, upper arm, elbow, forearm, wrist, hand, hip, thigh, knee, leg, ankle, and foot. Multiple sites could be selected, or “no pain” could be indicated.

3. Morbidities

Participants were asked to report morbidities from a predefined list. Options included hypertension, diabetes, asthma, anaemia, dyslipidaemia, vision impairment, hearing impairment, urinary incontinence, chronic kidney disease, chronic obstructive pulmonary disease, coronary artery disease, osteoarthritis, rheumatoid arthritis, osteoporosis, cancer, neurological disease (e.g., stroke, multiple sclerosis), history of serious fractures (e.g., hip or femur), and major surgery (e.g., total knee replacement). For analytic purposes, we created a combined “chronic musculoskeletal condition” variable, coded as present if participants reported ≥1 of the following self-reported diagnoses: osteoarthritis, rheumatoid arthritis, osteoporosis, history of serious fractures (e.g., hip or femur), or major joint surgery (e.g., total knee replacement).

### 2.6 Sample size calculation

Primary analyses used multivariable logistic regression with binary outcomes (site-specific musculoskeletal pain and morbidities). Sample adequacy was determined using the events-per-variable (EPV) criterion (≥10 events per estimated parameter; conservative target ≥20), which is widely recommended for logistic models (26, 27). Each adjusted model included 5–6 parameters (age, sex, smoking status, and BMI with two indicator terms). Hence, the minimum number of outcome events required per model was ≥50–120 (EPV 10–20). With a total sample of *N* = 298, outcomes with a prevalence of ≥17% (EPV ≈ 10) to ≥34% (EPV ≈ 20) met these thresholds, indicating that adequate power was available for common outcomes (e.g., prevalent musculoskeletal pain sites and common morbidities). Conversely, models for rare outcomes (low-prevalence sites or conditions) were underpowered. To support the EPV assessment, a *post hoc* power calculation was performed for logistic regression (Wald test) with an assumed medium effect (OR ≈ 1.8), *α* = 0.05, and five predictors. The reference to the Wald test here relates to the *post hoc* power calculation in the primary analyses, overall model significance was evaluated using likelihood-ratio (Omnibus *χ*^2^) tests, with Wald statistics used only for testing individual regression coefficients. Under outcome prevalences in the 0.20–0.35 range identified in the dataset, the estimated power with *N* = 298 was approximately 0.87–0.96. For prevalences near 0.10, power fell below 0.80, consistent with the EPV assessment. On this basis, the study was considered adequately powered for common outcomes and underpowered for rare outcomes.

### 2.7 Statistical analysis

All statistical analyses were conducted using IBM SPSS Statistics, version 27 (IBM Corp., Armonk, NY, USA). A *p* < 0.05 was considered statistically significant. Descriptive statistics were used to summarize sample characteristics. Categorical variables (e.g., sex, BMI, smoking status, comorbidities, and presence of musculoskeletal pain at each site) were summarized as frequencies and percentages, while continuous variables (age, BMI) were summarized as means and standard deviations. Group comparisons were performed using chi-square tests for categorical variables and independent-samples *t*-tests for continuous variables.

Multivariable logistic regression analyses were used to estimate predictors of musculoskeletal pain outcomes. Binary logistic regression model was run for all sites of musculoskeletal pain, with age, sex, BMI, and smoking status entered as independent variables. To assess musculoskeletal pain burden, a multisite pain variable was created (0 = 0–1 site, 1 = ≥2 sites) and analysed using the same predictors. Similarly, multivariable logistic regression models were run for morbidities with sufficient case counts to support stable estimation. (e.g., hypertension, diabetes, dyslipidaemia). Interaction analyses were conducted to explore whether the associations between predictors and outcomes differed across subgroups (e.g., smoking × BMI, age × sex). Interaction terms were added to logistic regression models, and stratified analyses were performed when significant interactions were detected. Model diagnostics included evaluation of multicollinearity, pseudo R^2^ indices, and Hosmer–Lemeshow goodness-of-fit tests. Odds ratios (ORs) with 95% confidence intervals (CIs) were reported for each model to indicate the magnitude and direction of associations.

## 3 Results

### 3.1 Sociodemographic characteristics and prevalence of musculoskeletal pain and morbidities

**Figure 1:**
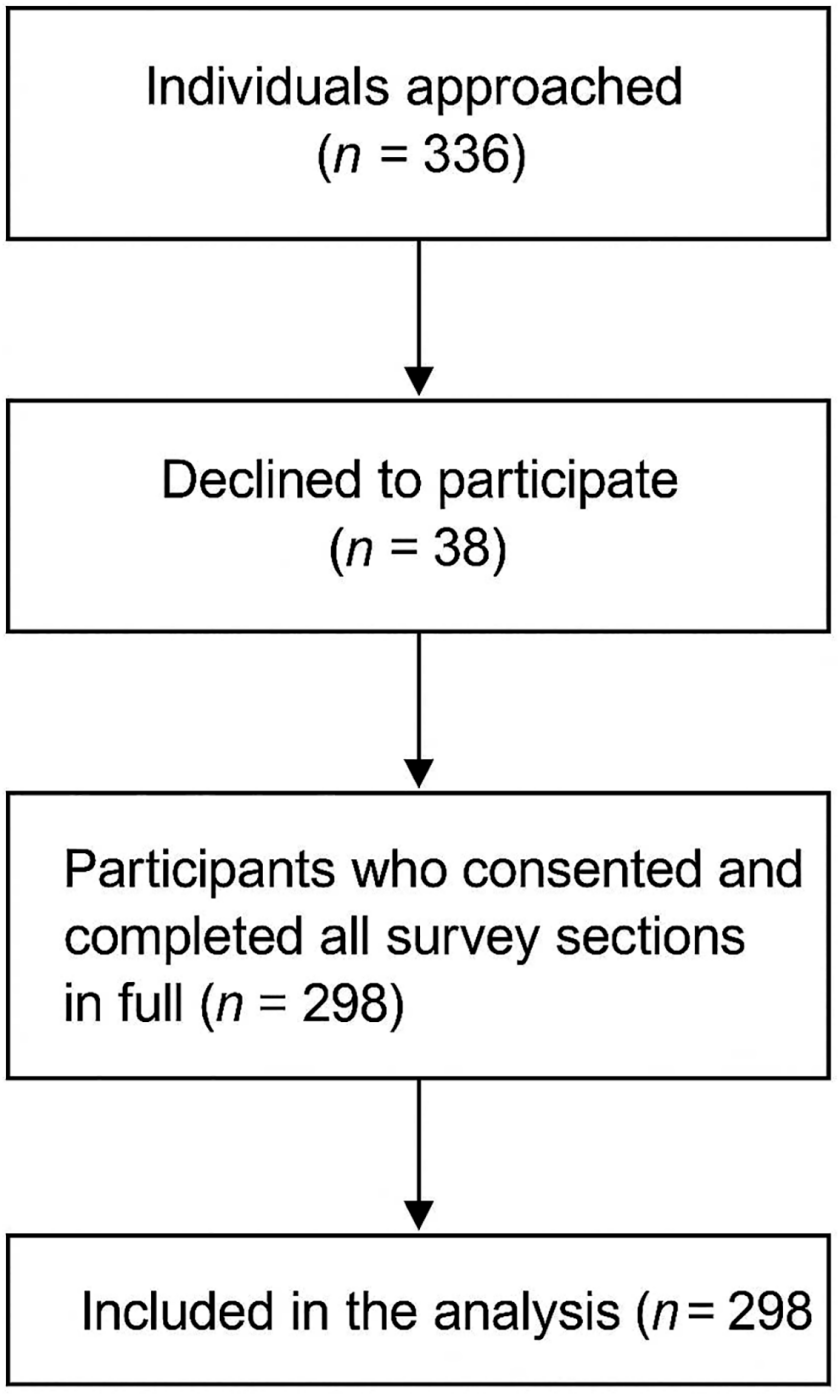
Flow chart of the study.

A total of 336 individuals were approached, of whom 38 declined to participate. The final sample comprised 298 participants who provided informed consent and completed all survey sections in full (Figure 1). The mean age of participants was 58.2 ± 6.3 years. Nearly half were overweight (47.0%) and one-third were obese (32.9%). Most lived with family (94.6%), were married (88.6%), and were retired or unemployed (70.8%). Table 1 summarizes the sociodemographic characteristics of the participants.

**Table 1:** Sociodemographic characteristics of the participants.

Variable	Category	*N* (%)
Age	Mean ± SD (range)	58.2 ± 6.3 (50–85)
BMI categories	Normal	60 (20.1%)
	Overweight	140 (47.0%)
	Obesity	98 (32.9%)
Living arrangement	With family/roommates	282 (94.6%)
	Alone	16 (5.4%)
Marital status	Married/Engaged	264 (88.6%)
	Single/Widowed/Separated/Divorced	34 (11.4%)
Education	High school or less	77 (25.8%)
	Bachelor/Diploma	141 (47.3%)
	Higher (Master’s/Doctoral)	80 (26.8%)
Employment	Working	87 (29.2%)
	Retired/Unemployed	211 (70.8%)
Primary work environment	Office-based	167 (56.0%)
	Field-based	131 (44.0%)
Smoking	No smoking	272 (91.3%)
	Current smoker	26 (8.7%)

N, number; %: percentage; BMI, body mass index; SD, standard deviation.

Musculoskeletal pain at any site was highly prevalent, affecting 73.2% of participants, with the low back (30.9%), knee (26.2%), neck (23.8%), and shoulder (21.8%) were the most frequent sites of musculoskeletal pain (Table 2). For morbidities, hypertension (29.5%) and diabetes (28.2%) were the most common, followed by dyslipidaemia (14.4%); nearly one-quarter of participants (24.5%) reported multimorbidity (≥2 conditions) (Table 3).

**Table 2:** Distribution of musculoskeletal pain by anatomical site among participants.

Variable	*N* (%)
Any musculoskeletal pain (≥1 site)	218 (73.2%)
Any musculoskeletal pain (≥2 site)	61(20.5%)
Low back	92 (30.9%)
Knee	78 (26.2%)
Neck	71 (23.8%)
Shoulder	65 (21.8%)
Foot	42 (14.1%)
Headache	31 (10.4%)
Thoracic	31 (10.4%)
Hip	14 (4.7%)
Hand	14 (4.7%)
Leg	17 (5.7%)
Thigh	16 (5.4%)
Ankle	16 (5.4%)
Abdomen	22 (7.4%)
Upper arm	21 (7.0%)
Forearm	11 (3.7%)
Wrist	7 (2.3%)
Pelvic	8 (2.7%)

N, number; %: percentage.

**Table 3:** Prevalence of morbidity and musculoskeletal pain by age group.

Variable	*N* (%)			*p*-value
	50–60 years (*n* = 218)	≥61 years (*n* = 80)	Total (i = 298)	
Morbidity condition				
Multimorbidity (≥2)	49 (22.5%)	24 (30.0%)	73 (24.5%)	0.181
Chronic musculoskeletal condition	11 (5.0%)	5 (6.2%)	16 (5.4%)	0.683
Osteoporosis	6 (2.8%)	7 (8.8%)	13 (4.4%)	**0.025**
Arthritis	12 (5.5%)	6 (7.5%)	18 (6.0%)	0.522
Dyslipidaemia	28 (12.8%)	15 (18.8%)	43 (14.4%)	0.199
Anaemia	6 (2.8%)	3 (3.7%)	9 (3.0%)	0.656
Asthma	11 (5.0%)	3 (3.7%)	14 (4.7%)	0.639
Diabetes Mellitus	52 (23.9%)	32 (40.0%)	84 (28.2%)	**0.006**
Hypertension	61 (28.0%)	27 (33.8%)	88 (29.5%)	0.333
Musculoskeletal pain site				
Headache	28 (12.8%)	3 (3.8%)	31 (10.4%)	**0.023**
Neck	55 (25.2%)	16 (20.0%)	71 (23.8%)	0.348
Thoracic	22 (10.1%)	9 (11.3%)	31 (10.4%)	0.772
Low back	71 (32.6%)	21 (26.3%)	92 (30.9%)	0.295
Sacroiliac	4 (1.8%)	0 (0.0%)	4 (1.3%)	0.223
Pelvic	8 (3.7%)	0 (0.0%)	8 (2.7%)	0.082
Abdomen	19 (8.7%)	3 (3.8%)	22 (7.4%)	0.146
Shoulder	51 (23.4%)	14 (17.5%)	65 (21.8%)	0.275
Upper arm	16 (7.3%)	5 (6.3%)	21 (7.0%)	0.745
Elbow	5 (2.3%)	1 (1.3%)	6 (2.0%)	0.570
Forearm	8 (3.7%)	3 (3.8%)	11 (3.7%)	0.974
Wrist	6 (2.8%)	1 (1.3%)	7 (2.3%)	0.448
Hand	9 (4.1%)	5 (6.3%)	14 (4.7%)	0.443
Hip	12 (5.5%)	2 (2.5%)	14 (4.7%)	0.277
Thigh	15 (6.9%)	1 (1.3%)	16 (5.4%)	0.056
Knee	57 (26.1%)	21 (26.3%)	78 (26.2%)	0.986
Leg	14 (6.4%)	3 (3.8%)	17 (5.7%)	0.378

N, number; %: percentage. *p-*values were derived from chi-square tests, and statistically significant differences between age groups (*p* < 0.05) are presented in bold.

### 3.2 Age-group differences in the prevalence of musculoskeletal pain and morbidities

Chi-square tests showed that osteoporosis (*p* = 0.025) and diabetes mellitus (*p* = 0.006) were significantly more prevalent among participants aged ≥61 years compared with 50–60 years. No significant age-group differences were observed for other examined variables (Table 3).

For musculoskeletal pain, headache pain was more common (*p* = 0.023) in the younger group (50–60 years: 12.8%) than in the older group (≥61 years: 3.8%). No other sites of musculoskeletal showed significant age-group differences (all *p* > 0.05).

### 3.3 Site-specific predictors of musculoskeletal pain

Multivariable logistic regression analyses were used to explore demographic and lifestyle predictors of site-specific musculoskeletal pain. Model calibration was acceptable for most sites of musculoskeletal pain (Hosmer–Lemeshow *p* > 0.05), except for leg pain (*p* = 0.018), indicating overall acceptable fit. As several musculoskeletal pain sites had low frequencies, sensitivity at the default 0.50 cutoff was low; interpretation therefore focused on ORs and 95% CIs.

Female sex was the strongest and most consistent predictor, with significantly higher odds of musculoskeletal pain at the wrist (OR ≈ 16.6), hand (OR ≈ 9.6), upper arm (OR ≈ 7.2), headache (OR ≈ 3.8), neck (OR ≈ 2.6), shoulder (OR ≈ 3.0), low back (OR ≈ 2.8), leg (OR ≈ 3.9), and foot (OR ≈ 3.0). Age showed inverse associations with headache pain (OR = 0.91 per year), abdominal pain (OR = 0.91 per year), and thigh pain (OR = 0.89 per year). Complete model outputs are presented in Table 4 after adjustments for age, BMI, sex and smoking status.

**Table 4:** Adjusted logistic regression models for predictors of musculoskeletal pain sites.

Musculoskeletal pain site		Predictors				
		Age	Sex (Female vs. Male)	BMI (Overweight vs. Normal)	BMI (Obesity vs. Normal)	Smoking (Yes vs. No)
Any pain	OR (95% CI)	0.98 (0.94–1.02)	**3.10 (1.73–5.53)**	1.07 (0.57–2.00)	1.80 (0.92–3.53)	0.76 (0.32–1.77)
	*P*-value	0.314	**<0.001**	0.841	0.085	0.519
Headache	OR (95% CI)	**0.91 (0.84–0.99)**	**3.83 (1.73–8.52)**	0.79 (0.27–2.31)	1.32 (0.45–3.85)	2.63 (0.86–8.01)
	*P*-value	**0.019**	**0.001**	0.666	0.614	0.089
Neck	OR (95% CI)	0.99 (0.95–1.04)	**2.58 (1.44–4.63)**	1.23 (0.59–2.57)	1.10 (0.50–2.42)	0.80 (0.28–2.25)
	*P*-value	0.687	**0.002**	0.585	0.811	0.671
Thoracic	OR (95% CI)	1.02 (0.96–1.08)	**2.64 (1.20–5.83)**	4.28 (0.95–19.37)	4.18 (0.89–19.57)	0.76 (0.17–3.49)
	*P*-value	0.500	**0.016**	0.059	0.069	0.725
Low back	OR (95% CI)	0.98 (0.93–1.02)	**2.83 (1.60–5.00)**	0.68 (0.34–1.37)	1.78 (0.88–3.60)	0.54 (0.19–1.55)
	*P*-value	0.253	**<0.001**	0.280	0.111	0.254
Pelvic	OR (95% CI)	0.91 (0.78–1.06)	2.56 (0.59–11.06)	— †	— †	— †
	*P*-value	0.204	0.209			
Abdomen	OR (95% CI)	**0.91 (0.84–0.99)**	1.90 (0.76–4.76)	0.92 (0.27–3.17)	1.33 (0.38–4.62)	0.46 (0.06–3.63)
	*P*-value	**0.048**	0.172	0.896	0.653	0.459
Shoulder	OR (95% CI)	0.97 (0.93–1.02)	**2.99 (1.64–5.44)**	1.52 (0.68–3.41)	1.53 (0.66–3.59)	0.66 (0.22–2.05)
	*P*-value	0.244	**<0.001**	0.311	0.323	0.475
Upper arm	OR (95% CI)	1.00 (0.92–1.08)	**7.21 (2.71–19.15)**	1.84 (0.47–7.19)	1.55 (0.37–6.59)	— †
	*P*-value	0.942	**<0.001**	0.383	0.550	
Forearm	OR (95% CI)	1.01 (0.92–1.11)	2.68 (0.77–9.36)	2.84 (0.33–24.39)	2.58 (0.28–23.91)	— †
	*P*-value	0.845	0.122	0.342	0.404	
Wrist	OR (95% CI)	0.93 (0.79–1.09)	**16.55 (1.90–144.29)**	0.90 (0.08–10.65)	2.58 (0.26–25.34)	— †
	*P*-value	0.342	**0.011**	0.932	0.417	
Hand	OR (95% CI)	1.04 (0.95–1.13)	**9.58 (2.83–32.47)**	1.69 (0.32–8.80)	1.63 (0.29–9.11)	1.02 (0.12–8.88)
	*P*-value	0.433	**<0.001**	0.535	0.581	0.988
Hip	OR (95% CI)	0.96 (0.87–1.07)	2.97 (0.98–8.99)	1.82 (0.37–9.01)	1.21 (0.21–6.96)	— †
	*P*-value	0.472	0.055	0.464	0.830	
Thigh	OR (95% CI)	**0.89 (0.80–0.99)**	2.79 (0.97–8.01)	0.54 (0.16–1.83)	0.41 (0.10–1.66)	0.70 (0.08–5.75)
	*P*-value	**0.048**	0.057	0.324	0.214	0.737
Knee	OR (95% CI)	1.00 (0.96–1.04)	1.26 (0.69–2.28)	1.18 (0.57–2.45)	1.67 (0.79–3.54)	0.35 (0.10–1.22)
	*P*-value	0.931	0.456	0.656	0.183	0.100
Leg	OR (95% CI)	0.97 (0.89–1.06)	**3.93 (1.41–10.92)**	0.81 (0.19–3.43)	1.64 (0.41–6.61)	2.99 (0.75–11.88)
	*P*-value	0.491	**0.009**	0.777	0.485	0.119
Foot	OR (95% CI)	0.95 (0.90–1.01)	**2.97 (1.48–5.95)**	1.02 (0.39–2.66)	1.69 (0.64–4.42)	1.68 (0.57–4.90)
	*P*-value	0.131	**0.002**	0.968	0.287	0.344

BMI: body mass index; OR: odds ratio; 95% CI: 95% confidence interval. ^†^Model instability due to non-convergence or quasi-complete separation of data. Statistically significant predictors are presented in bold (*p* < 0.05).

### 3.4 Predictors of morbidities

Older age was associated with osteoporosis (OR = 1.13), dyslipidaemia (OR = 1.06), hypertension (OR = 1.05), and diabetes (OR = 1.06). Female sex was associated with osteoporosis (OR = 8.71), dyslipidaemia (OR = 3.07), chronic musculoskeletal pain (OR = 5.43), arthritis (OR = 4.31), and anaemia (OR = 4.36). Obesity was associated with hypertension only (OR = 3.83). Full regression results are presented in Table 5.

**Table 5:** Adjusted logistic regression models for predictors of morbidities.

Morbidity condition		Predictors				
		Age	Sex (Female vs. Male)	BMI (Overweight vs. Normal)	BMI (Obesity vs. Normal)	Smoking (Yes vs. No)
Osteoporosis	OR (95% CI)	**1.13 (1.05–1.23)**	**8.71 (2.43–31.23)**	3.22 (0.35–29.95)	4.29 (0.46–40.08)	2.89 (0.50–16.77)
	P value	**0.002**	**0.001**	0.305	0.202	0.238
Dyslipidaemia	OR (95% CI)	**1.06 (1.01–1.12)**	**3.07 (1.52–6.21)**	1.91 (0.71–5.11)	1.73 (0.61–4.85)	0.55 (0.12–2.53)
	P value	**0.013**	**0.002**	0.201	0.301	0.445
Hypertension	OR (95% CI)	**1.05 (1.01–1.10)**	0.87 (0.47–1.61)	1.89 (0.86–4.14)	**3.83 (1.72–8.52)**	1.54 (0.64–3.72)
	P value	**0.017**	0.656	0.113	**0.001**	0.341
Diabetes	OR (95% CI)	**1.06 (1.02–1.11)**	0.58 (0.30–1.12)	1.86 (0.90–3.84)	1.36 (0.62–2.96)	0.80 (0.30–2.13)
	P value	**0.005**	0.107	0.096	0.440	0.659
Chronic MSK pain	OR (95% CI)	1.06 (0.98–1.14)	**5.43 (1.80–16.37)**	— †	— †	— †
	P value	0.151	**0.003**			
Arthritis	OR (95% CI)	1.07 (1.00–1.15)	**4.31 (1.52–12.23)**	1.26 (0.23–6.87)	4.24 (0.87–20.63)	— †
	P value	0.051	**0.006**	0.793	0.073	
Anaemia	OR (95% CI)	1.05 (0.95–1.16)	**4.36 (1.08–17.58)**	0.45 (0.11–1.92)	0.15 (0.02–1.38)	— †
	P value	0.389	**0.039**	0.281	0.093	
Asthma	OR (95% CI)	1.02 (0.94–1.10)	2.49 (0.81–7.66)	0.43 (0.08–2.20)	1.71 (0.43–6.77)	0.98 (0.12–8.11)
	P value	0.702	0.112	0.308	0.446	0.982

OR: odds ratio; 95% CI: 95% confidence interval; MSK: musculoskeletal. ^†^Model instability due to quasi-complete separation of data. Statistically significant predictors are presented in bold (*p* < 0.05).

### 3.5 Interaction effects on morbidity outcomes

Three significant interactions were identified. For hypertension, a significant smoking × BMI interaction was found (OR = 0.22), suggesting that smoking increased hypertension risk most strongly among normal-weight participants and was attenuated in overweight and obese groups. For diabetes, two interactions reached significance: age × hypertension (OR = 1.02), showing that the age-related increase in diabetes risk was higher among participants with hypertension, and hypertension × BMI (OR = 1.59), showing that the strength of the hypertension–diabetes association differed across BMI categories. Full interaction model results are presented in Table 6.

**Table 6:** Interaction effects of demographic and clinical predictors on morbidity outcomes.

Outcome	Interaction term	B (SE)	OR (95% CI)	*P*-value	
Hypertension	Age × Sex	0.03 (0.05)	1.03 (0.93–1.15)	0.537	
	Age × BMI	0.00 (0.03)	1.00 (0.95–1.06)	0.928	
	Age × Diabetes	−0.02 (0.04)	0.98 (0.90–1.06)	0.604	
	Age × Smoking	−0.01 (0.08)	0.99 (0.84–1.17)	0.940	
	Smoking × Sex	−1.09 (1.32)	0.34 (0.03–4.48)	0.410	
	Smoking × BMI	−1.50 (0.69)	0.22 (0.06–0.86)	**0.029**	
	Smoking × Diabetes	−0.26 (1.01)	0.77 (0.11–5.61)	0.798	
Diabetes	Hypertension × BMI	0.47 (0.13)	1.59 (1.24–2.05)	**<0.001**	
	Age × Sex	0.05 (0.06)	1.05 (0.94–1.17)	0.435	
	Age × BMI	−0.04 (0.03)	0.96 (0.91–1.02)	0.198	
	Age × Smoking	0.04 (0.09)	1.05 (0.87–1.26)	0.640	
	Age × Hypertension	0.02 (0.01)	1.02 (1.01–1.03)	**<0.001**	

B: regression coefficient; SE: standard error; OR: odds ratio; 95% CI: 95% confidence interval. Statistically significant interaction effects are presented in bold (*p* < 0.05).

### 3.6 Interaction effects on musculoskeletal pain outcomes

Significant sex × BMI effects were observed for shoulder pain (OR = 13.53, 95% CI: 1.85–98.76) and knee pain (OR = 7.87, 95% CI: 1.18–52.63), with obese females showing markedly higher odds compared with normal-weight males. Overall, obesity in females was strongly linked to increased risk of shoulder and knee pain, but not for other sites. Full model details are presented in Table 7.

**Table 7:** Interaction effects of demographic and clinical predictors on musculoskeletal pain outcomes.

Outcome	Interaction term	B (SE)	OR (95% CI)	*P*-value
Neck pain	Sex × Overweight	−0.463 (0.701)	0.63 (0.16–2.43)	0.504
	Sex × Obese	0.312 (0.712)	1.37 (0.34–5.50)	0.668
Shoulder pain	Sex × Overweight	0.881 (0.691)	2.41 (0.62–9.32)	0.196
	Sex × Obese	2.605 (1.009)	13.53 (1.85–98.76)	**0.010**
Knee pain	Sex × Overweight	0.662 (0.770)	1.94 (0.43–8.74)	0.370
	Sex × Obese	2.064 (0.967)	7.87 (1.18–52.63)	**0.033**
Foot pain	Sex × Overweight	−0.143 (0.767)	0.87 (0.19–4.00)	0.849
	Sex × Obese	0.268 (0.893)	1.31 (0.23–7.51)	0.765
Low back pain	Sex × Overweight	0.421 (0.655)	1.52 (0.42–5.45)	0.528
	Sex × Obese	0.678 (0.701)	1.97 (0.50–7.71)	0.325
Multisite pain (BMI)	Sex × Overweight	0.412 (0.420)	1.51 (0.66–3.46)	0.323
	Sex × Obese	0.331 (0.430)	1.39 (0.60–3.21)	0.438
Multisite pain (High BMI vs Normal)	Sex × High BMI	1.200 (0.820)	3.32 (0.69–15.99)	0.135

B: regression coefficient; SE: standard error; OR: odds ratio; 95% CI: 95% confidence interval; BMI: body mass index. Statistically significant interaction effects are presented in bold (*p* < 0.05).

## 4 Discussion

This study investigated the prevalence and predictors of musculoskeletal pain and common morbidities among adults aged ≥50 years in Saudi Arabia, with an emphasis on identifying prespecified interaction effects between demographic and clinical variables. Musculoskeletal pain was highly prevalent, affecting nearly 75% of participants, with the low back, knee, neck, and shoulder as the most frequently affected sites. Hypertension and diabetes were the most frequently reported morbidities, observed in approximately one-third (33%) of participants, while one quarter (25%) of the participants had multimorbidity (≥2 conditions). Multivariable logistic regression analyses identified female sex as the strongest and most consistent predictor of musculoskeletal pain across multiple anatomical regions examined, while obesity was most strongly associated with hypertension. Importantly, interaction analyses indicated that the risk of hypertension from smoking was greatest in individuals with higher BMI, that the effect of age on diabetes risk was greater among participants with hypertension and higher BMI, and that obese females exhibited substantially higher odds of shoulder and knee pain compared with normal-weight males. These findings underscore the substantial burden of musculoskeletal pain and chronic conditions in older adults in Saudi Arabia and highlight the importance of examining effect modification to identify high-risk subgroups that may be masked in main-effects models.

### 4.1 Predictors of musculoskeletal pain

In our sample of adults aged ≥50 years, musculoskeletal pain was highly prevalent, with the low back, knee, neck, and shoulder being the most commonly affected sites. This prevalence is higher than the national estimate for adults across age groups where chronic pain prevalence was 46% which aligns with the observed trend of increasing pain with age (28). Chronic back pain was also the most frequently reported pain site in Saudi adults, consistent with the distribution observed in our cohort (17). The prevalence of low back pain in our study aligns with the global musculoskeletal pain burden, more than 0.5 billion individuals in 2020 and thereby underscoring its major contribution to musculoskeletal pain among older adults (1). Across the Gulf Cooperation Council (GCC) countries, including Saudi Arabia, musculoskeletal pain rank among the primary causes of disability, with a 2019 age-adjusted prevalence of 19%, in line with established baseline rates, suggesting that older adults may exhibit even greater symptom severity (18).

A high concurrence of hypertension and diabetes was identified among participants, and this pattern is also reflected in national epidemiology (29). National surveys and systematic reviews report substantial hypertension prevalence with pronounced age-related increases (e.g., ~48–55% among those ≥65 years) (30, 31). Similarly, diabetes remains highly prevalent in Saudi society alongside obesity; the 2024/2025 International Diabetes Federation Diabetes Atlas estimates that 23% of Saudi adults (≈5.3 million) are living with diabetes, placing Saudi Arabia in the group of countries with the highest prevalence (32). Obesity prevalence in adults is similarly among the highest worldwide (~38%), as reported in national studies (20–39%). The high prevalence observed in the current study is consistent with both national and global evidence. Overall, cardiometabolic risk factors are often clustered due to the impact of common causes such as higher body fat, insulin resistance and physical inactivity (33). This provides context for the co-occurrence of hypertension and diabetes and the high prevalence observed in our cohort. Furthermore, obesity was strongly associated with hypertension than with musculoskeletal pain in the main-effects models. This pattern can be explained by the biomechanical impact of excess weight on weight-bearing joints, with musculoskeletal pain associations becoming more evident in the presence of effect modification identified in the interaction analyses.

Female sex was identified as the strongest and most consistent predictor of musculoskeletal pain across several anatomical sites (wrist/hand, neck, shoulder, low back, and leg). This pattern has been consistently noted in population-based studies, with females showing higher odds of single and multisite musculoskeletal pain than males, which may be driven by a combination of biological (e.g., hormonal and immune modulation, central sensitization), and psychosocial factors (34, 35). For less prevalent sites of musculoskeletal pain such as headache, abdominal and thigh pain, age was inversely associated with pain occurrence. Although epidemiology generally shows higher overall musculoskeletal pain with advancing age, site-specific profiles often change in older adults due to treatment side effects (e.g., long-term analgesic use), and selective reporting (36, 37). Hence, the inverse relationships noted for these sites do not oppose the general age-related trends in pain burden but rather indicate redistribution of affected sites of musculoskeletal pain in older adults.

High BMI was not consistently associated with musculoskeletal pain across sites in the fully adjusted models, likely reflecting site specificity (mechanical stress is strongest at the knee/hip site and to a lesser extent the lumbar spine) and limited statistical power less common pain sites in our dataset (37). Large population studies link higher BMI to knee pain and to low-back pain via mechanical and inflammatory mechanisms; therefore, modest knee and low-back findings should be interpreted carefully and in the context of the significant sex × BMI interactions observed for shoulder and knee. Furthermore, smoking showed borderline associations, which align with variable evidence in the literature. Although smoking has been linked to chronic pain via pro-inflammatory and microvascular mechanisms, effects are often small, site-specific, and modified by additional factors (e.g., BMI, sex) (38). In our study, the association between smoking and pain appears better explained by effect modification than by a consistent main effect.

### 4.2 Predictors of morbidities (hypertension and diabetes)

In our data, older age and higher BMI were strongly associated with hypertension, while sex and smoking were not significant after adjustment. This pattern is in line with international evidence that hypertension rises sharply with age and is more frequent in overweight individuals (39). Furthermore, the Saudi Health Interview Survey reported higher hypertension risk associated with older age, obesity and diabetes (40). Some variability exists across Saudi studies: a multicentre cohort in Tabuk reported age, male sex, smoking, and physical inactivity as predictors of hypertension among clinical populations (41). This is contrast to our null smoking and sex effects, and likely reflects variations in settings (hospital vs. community), age distribution and covariates adjustment. Higher odds of diabetes were observed with older age and greater adiposity in our survey, consistent with recent Saudi syntheses. A meta-analysis reported high adult diabetes prevalence and higher risk with advancing age and higher BMI, closely matching our findings (42). Roughly 23% of Saudi adults (5.3 million) are estimated to have diabetes, underscoring the significance of the observed age/BMI associations. Minor discrepancies across single-site Saudi studies are likely due to differences in sampling frames and adjustment sets.

### 4.3 Interaction effects

In the current survey, the association between smoking and hypertension was not heterogeneous across BMI categories. In comparable cohorts, evidence suggests that BMI modifies the association between smoking and incident hypertension (43). For example, a 22-year prospective Chinese cohort study showed that incident hypertension was predicted by current smoking mainly in normal-weight adults, with little evidence of an effect among overweight and obese individuals. Smoking and high BMI have been reported to be associated with hypertension but effect-modification patterns vary across studies, likely reflecting differences in age structure, BMI thresholds, and covariate adjustment sets (44) Taken together, our finding of BMI-dependent smoking effects is consistent with the broader literature on effect modification, while the specific stratum with the largest effect may vary across cohorts.

Interaction effects involving age, hypertension, BMI, and diabetes showed that the age-related increase in the odds of diabetes was more pronounced among individuals with hypertension, and diabetes risk was greatest when hypertension co-occurred with elevated BMI. These patterns are consistent with large-scale evidence showing that hypertension independently predicts new-onset type 2 diabetes and that its incidence is reduced by blood pressure-lowering therapy (45). Recent evidence also demonstrates a synergistic interaction between high BMI and hypertension on diabetes risk (e.g., a combined risk exceeding the sum of individual effects) (46).

Interaction effects involving sex, BMI, and musculoskeletal pain showed that obese females had higher odds of pain at the shoulder and knee compared with normal-weight males. For the knee, this pattern is supported by epidemiological studies reporting that the association between obesity and knee osteoarthritis/pain is more pronounced in females (47, 48). Contemporary reviews also emphasize sex differences in osteoarthritis prevalence and pain, with women generally at higher risk and BMI contributing more strongly to risk in women than men in several cohorts. For the shoulder, high BMI and metabolic factors have been associated with shoulder pain, but sex-by-BMI interactions are not consistently documented; thus; the current finding extends prior observations and identifies a subgroup that may benefit from targeted prevention.

Across outcomes, the interaction patterns indicate that high-risk subgroups can be identified via risk stratification using simple attributes (sex, BMI, smoking) that are often overlooked in main-effects models. Apparent discrepancies are likely attributable to design variations (e.g., age range, BMI thresholds, and modelling choices) rather than substantive contradictions, which is an expected feature of interaction estimates in survey data. Replication in larger, multi-regional Saudi samples using standardized covariates will help determine which strata are most appropriate for targeted screening and prevention.

### 4.4 Limitations and future directions

This study has several limitations. First, the cross-sectional design prevents causal inference and the time-ordering of musculoskeletal pain and morbidities. Second, the online convenience sample likely underrepresents older adults with limited digital access, thereby constraining generalizability beyond community-dwelling adults ≥50 years. Third, key variables were self-reported (pain sites, height, weight), introducing potential recall and misclassification biases (e.g., BMI error from self-report; musculoskeletal pain site misattribution). Fourth, pain was recorded as presence/absence at each site over a short recall period, without standardized measures of severity (e.g., VAS/NRS) or duration; therfore, we could not distinguish acute from chronic pain or stratify by pain intensity or impact. Fifth, several outcomes were low prevalence, resulting in limited events per variable and occasional non-convergence/quasi-separation in logistic models. Estimates for these rarer sites/conditions require cautious interpretation. Finally, despite adjustment for key confounders, yet residual confounding may persist (e.g., physical activity, analgesic use or other medications). Future studies should use random sampling, validated and standardized pain instruments (severity, duration, interference), and longitudinal follow-up to clarify temporal relationships.

### 4.5 Clinical implications and recommendations

Musculoskeletal pain in adults ≥50 frequently coexists with hypertension and/or diabetes; thus, each clinical encounter for musculoskeletal pain should include brief cardiometabolic checks (e.g., blood pressure, blood glucose, BMI, smoking status). For knee, hip and low back pain, prioritize non-pharmacologic management (e.g., structured exercise and/or other physiotherapy approaches, patient education, and weight management). Simple risk stratification (sex, BMI, smoking) can be used to tailor counselling, with weight reduction and strengthening emphasised for females with shoulder or knee pain and smoking-cessation support targeted to smokers with hypertension. Finally, multisite musculoskeletal pain (≥2 sites) should warrant comprehensive assessment, including sleep, mood, and physical activity, and the development of coordinated multidisciplinary care plans.

## 5 Conclusion

In this community-based study of adults aged ≥50 years in Saudi Arabia, musculoskeletal pain was highly prevalent particularly at the low back, knee, neck, and shoulder and was accompanied by considerable burdens of hypertension and diabetes. Female sex was the strongest predictor of musculoskeletal pain across multiple sites, while obesity was more strongly associated with hypertension than with widespread musculoskeletal pain. Importantly, interaction analyses revealed heterogeneity that may be obscured by main-effects models: the smoking-hypertension association varied by BMI (strongest at normal weight), the age-related increase in diabetes risk was heightened among individuals with hypertension and further increased at higher BMI, and obese females had markedly higher odds of shoulder and knee pain.

These findings have several implications for care: musculoskeletal consultations should include brief cardiometabolic screening, prioritize non-pharmacological management for knee, hip and low back pain, and use simple stratifiers (e.g., sex, BMI, smoking) to guide counselling and follow-up. Multisite musculoskeletal pain should prompt comprehensive assessment and coordinated, multidisciplinary support. Considering the cross-sectional design, self-reported measures and convenience sampling, causal inference is limited, and some estimates lack precision for rarer outcomes. Future research should use representative, random sampling, standardized musculoskeletal pain assessment tools, and prospective longitudinal designs to confirm effect-modification patterns. Integrating musculoskeletal and cardiometabolic prevention into primary care may advance Saudi Arabia’s Vision 2030 goals by reducing disability and extending healthy life expectancy.

## Data Availability

The raw data supporting the conclusions of this article will be made available by the authors, without undue reservation.

## Footnotes

1
www.surveymonkey.com
